# ANO1 (TMEM16A) in pancreatic ductal adenocarcinoma (PDAC)

**DOI:** 10.1007/s00424-014-1598-8

**Published:** 2014-08-28

**Authors:** D. R. P. Sauter, I. Novak, S. F. Pedersen, E. H. Larsen, E. K. Hoffmann

**Affiliations:** 1 Section for Cell and Developmental Biology, Department of Biology, University of Copenhagen, August Krogh Building, Universitetsparken 13, 2100 Copenhagen Ø, Denmark; 2 Section for Molecular Integrative Physiology, Department of Biology, University of Copenhagen, August Krogh Building, Universitetsparken 13, 2100 Copenhagen Ø, Denmark

**Keywords:** Cl^−^ channel, TMEM16A, VRAC, LRRC8, Cancer, Proliferation, Migration, Pancreas

## Abstract

Pancreatic ductal adenocarcinoma (PDAC) has one of the worst survival rates of all cancers. ANO1 (TMEM16A) is a recently identified Ca^2+^-activated Cl^−^ channel (CaCC) that is upregulated in several tumors. Although ANO1 was subject to extensive studies in the recent years, its pathophysiological function has only been poorly understood. The aim of the present study is to establish the significance of ANO1 in PDAC behavior and demarcate its roles in PDAC from those of the volume-regulated anion channel (VRAC). We performed qPCR and Western blot measurements on different PDAC cell lines (Panc-1, Mia PaCa 2, Capan-1, AsPC-1, BxPC-3) and compared the results to those obtained in a human pancreatic ductal epithelium (HPDE) cell line. All cancer cell lines showed an upregulation of ANO1 on mRNA and protein levels. Whole-cell patch-clamp recordings identified large Ca^2+^ and voltage-dependent Cl^−^ currents in PDAC cells. Using siRNA knockdown of ANO1 and three ANO1 inhibitors (T16A_inh_-A01, CaCC_inh_-A01, and NS3728), we found that ANO1 is the main constituent of CaCC current in PDAC cells. We further characterized these three inhibitors and found that they had unspecific effects on the free intracellular calcium concentration. Functional studies on PDAC behavior showed that surprisingly inhibition of ANO1 did not influence cellular proliferation. On the other hand, we found ANO1 channel to be pivotal in PDAC cell migration as assessed in wound healing experiments.

**Electronic supplementary material:**

The online version of this article (doi:10.1007/s00424-014-1598-8) contains supplementary material, which is available to authorized users.

## Introduction

Pancreatic ductal adenocarcinoma (PDAC) has one of the worst prognoses of all cancers, with a 5-year survival rate of less than 5 %. Incidence of PDAC correlates with increasing age and therefore is an increasing problem as world population is aging [16]. Dysfunction of ion channels is an emerging field in cancer research and differential expression of multiple classes of ion channels has been reported for different cancer tissues [20–22, 28, 29, 31, 35, 43]. Plasma membrane ion channels are involved in apoptosis [19], differentiation, and proliferation [17, 28], and are further associated with cell migration [15, 38] and cell volume regulation [14]. The latter is a crucial factor in regulation of cell proliferation (see [14]) and apoptosis [33], key processes that are usually deregulated in cancer [11].

In epithelia, including pancreatic ducts, Ca^2+^-activated Cl^−^ channel (CaCC) is important for secretory processes [27]. One of the favored molecular candidates for CaCC is the recently identified ANO1 (also known as TMEM16A, TAOS2, ORAOV2, and DOG1) [4, 9, 37, 48]. ANO1 is located on the amplicon 11q13 that is frequently amplified in human cancers with poor prognosis [1, 7, 26, 46]. ANO1 was shown to be upregulated in several cancer tissues including head and neck squamous cell carcinoma (HNSCC) and prostate-, breast-, and pancreatic cancer [1, 2, 22, 23, 45]. However, the physiological function of ANO1 has been found to vary among different cancer tissues. A pro-proliferative role of ANO1 was reported for breast- and prostate cancer [3, 22, 23], but this was not confirmed in HNSCC cells [1, 35]. Contradictory results are reported in the pancreatic cancer cell line CFPAC-1. Thus, Ruiz et al. [35] found a slight anti-proliferative effect of ANO1 using a knockdown and an overexpression strategy, whereas Mazzone et al. [23] showed a decrease in proliferation after application of the recently developed ANO1-specific inhibitor T16A_inh_-A01 (10 μM) [5, 23, 35]. ANO1 was further associated with cellular attachment, spreading, detachment, and invasion [1, 15]. All these studies used cell lines highly overexpressing ANO1 or with knockdown of ANO1 to assess its pathophysiological function, but suffered from the lack of a normal pancreatic epithelial cell control system for comparison.

A volume-regulated anion channel (VRAC), which is involved in restoring cell volume upon external osmotic perturbation, has been studied in several types of mammalian cells [14]. Synthesis of high-affinity Cl^−^ channel inhibitors, such as NS3728 [12], has facilitated insight in VRAC’s physiological role in cell cycle progression. A four-transmembrane protein, the Leucine-rich repeat-containing protein 8 (LRRC8), was recently identified as a main constituent of the VRAC current [32, 44].

The aim of the present study was to elucidate the role of ANO1 in PDAC behavior, compare it to a normal model cell line, and demarcate the roles of ANO1 from those of VRAC.

## Materials and methods

### Cell culture and transfection

All cell lines were grown at 37 °C and 5 % CO_2_. Immortalized human pancreatic ductal epithelium (HPDE) (H6c7 cell line, kind gift of Dr. M-S. Tsao from University Health Network in Toronto [8, 27]) cells were grown in keratinocyte serum-free medium supplemented by epidermal growth factor and bovine pituitary extract (Life Technologies, Inc., USA). Cells were passaged by gently trypsinization, subsequent neutralization with trypsin inhibitor (soybean, Life Technologies, Inc., USA), centrifugation (700 rpm/3 min), and resuspension. All cancer cells (obtained from ATCC, Germany) were passaged every 4–6 days by gently trypsinization. Panc-1 and Mia PaCa-2 were grown in Dulbecco’s Modified Eagle Medium with stable glutamine (DMEM/Biochrom, Germany), BxPC-3 and AsPC-1 in Roswell Park Memorial Institute medium with stable glutamine (RPMI 1640/Biochrom, Germany), and Capan-1 in Iscove’s Modified Dulbecco’s Medium (IMDM-1640/Biochrom, Germany), all were supplemented with 10 % *v*/*v* (20 % for Capan-1) Fetal Bovine Serum “Gold” (PAA Laboratories GmbH, Germany). Mia PaCa-2 growth medium was further supplemented with 2.5 % *v*/*v* horse serum (Biochrom, Germany). All cultures were further supplemented with 1 % *v*/*v* penicillin and streptomycin.

DharmaFECT 1 Transfection Reagent (Thermo Scientific, Germany) was used for transfection of siRNA targeting ANO1 (50 nM final concentration) or scrambled (5 nM final concentration). Cells were transfected according to the manufacturer’s protocol. Predesigned siRNA oligo was obtained from Sigma-Aldrich (5′-CCUUCAACGUCAGUGACUU[dT][dT]-3′, 5′-AAGUCACUGACGUUGAAGG[dT][dT]-3′) or negative control (Silencer® Negative Control No. 1 siRNA; Ambion, Denmark). ANO1 overexpressing HEK293 cells were generated by adding 0.5 μg/ml mANO1-GFP vector to DMEM containing 1 % *v*/*v* penicillin and streptomycin. The mixture was vortexed and incubated for 5 min, and 20 μg/ml polyethylenimine (PEI) was added. The mixture was vortexed again and added drop-wise to 60 % confluent HEK293 cells in DMEM medium containing 5 % *v*/*v* FBS and 1 % *v*/*v* penicillin and streptomycin after 10 min incubation at room temperature. Media was changed after 4 h incubation at 37 °C and 5 % CO_2_.

### Isolation of RNA, cDNA, and qPCR

Total RNA was extracted from cell cultures using Nucleo Spin II (MACHEREY-NAGEL, Germany). First strand complementary DNAs were synthesized using Superscript II (Invitrogen, Denmark) and Oligo-dTs following the manufacturer’s guidelines. PCR reaction mixtures were prepared using the FastStart Universal SYBR Green Master (Rox) mix (Roche, Denmark). Quantitative PCR experiments were carried out in triplicates. Primers used were as follows: ANO1-sense 5′-GCGTCCACATCATCAACATC-3′ and ANO1-antisense 5′-ATCCTCGTGGTAGTCCATCG-3′ [41]. ANO1 expression levels were normalized to the reference gene β-actin. The fold-change in gene expression was determined by the ΔΔC(t) method [36]. Data were expressed as expression relative to that in the control cell line HPDE.

### Electrophysiology

Cells were grown on poly-L-lysine coated coverslips. For knockdown experiments, cells were transfected with siRNA targeting ANO1 or scrambled siRNA shortly after complete attachment of the cells (approx. 3 h after plating). Currents were measured 36–48 h after transfection. Whole-cell patch-clamp recordings were performed using the Axopatch 200B amplifier interfaced to a Digidata 1440A controlled by pClamp10 software (Molecular Devices, USA). Analogue signals were acquired at 2.5 kHz and filtered at 1 kHz. Patch electrodes were pulled from borosilicate glass and had an input resistance of 2–6 MΩ, when filled with pipette solution (below). An agar bridge made of 3 % agar and 97 % of the bath solution containing NMDG-Cl (below) was used as reference electrode. Current activations were recorded from an output holding potential, *V*_out_ = 0 mV with ramp protocols spanning from −100 to 100 mV over 1 s and run continuously with 15-s intervals. Step protocols between −100 to 120 mV were generated from *V*_out_ = 0 mV with step increments of 20 mV and lengths of 2 s. Liquid junction potentials (*V*_lj_) were estimated as described in [10] with membrane potentials calculated as, *V*_m_ = *V*_out_ − *V*_lj_. Series resistance was compensated by 60–70 %. All recordings were performed at room temperature (18–23 °C). Instantaneous currents were measured 1 ms after each voltage step. The data was fitted using the Goldman–Hodgkin–Katz (GHK) current equation.

1 $$ {I}_{{\mathrm{Cl}}^{-}}= P\frac{z^2{F}^2 V}{RT}\frac{C_o-{C}_i{e}^{\frac{ z FV}{RT}}}{1-{e}^{\frac{ z FV}{RT}}} $$

Here, ${I}_{{\mathrm{Cl}}^{-}}$ is the current carried across the membrane by Cl^−^ ions, *P* is the permeability of the membrane for Cl^−^, *z* is the valence (−1), *F* is the Faraday constant, *V* is the membrane voltage, *R* is the universal gas constant, *T* is the absolute temperature, *C*_*o*_ and *C*_*i*_ are the extra- and intracellular concentration of Cl^−^, respectively. Steady-state permeability was calculated by solving Eq. 1 for *P*. The Boltzmann distribution (Eq. 2) was fitted to the permeabilities obtained above.

2 $$ {P}_{{\mathrm{Cl}}^{.}}={P}_{\max }+\frac{\left({P}_{\min }-{P}_{\max}\right)}{1+{e}^{\frac{V-{V}_{1/2}}{dV}}} $$

*V*_1/2_ is the voltage for half-maximal activation of the channels. The curves were normalized to *P*_min_ = 0 and *P*_max_ = 1.

### Solutions

One micromolar free Ca^2+^ pipette solution include the following (in mM): 100 CsAsp, 40 CsCl, 4.34 CaCl_2_, 4 Na_2_ATP, 1 MgCl_2_, 5 EGTA, 10 HEPES, pH 7.2, osmolality 295 mosmol/kg or 0 μM free Ca^2+^ solution containing no CaCl_2_ and 10 mM EGTA. Standard bath solution include the following (in mM): 150 NaCl, 1.5 CaCl_2_, 1 MgCl_2_, 10 glucose, 10 HEPES, pH 7.4, osmolality 315 mosmol/kg (adjusted with d-mannitol). Isotonic solution include the following (in mM): 85 NaCl, 2 MgCl_2_, 1.5 CaCl_2_, 10 glucose, 10 HEPES, pH 7.4, of osmolality 315 mosmol/kg (adjusted with d-mannitol). The hypotonic solution with osmolality of 210 mosmol/kg was obtained by omitting d-mannitol from the isotonic solution. For experiments testing efficiency of an inhibitor, NaCl was replaced by *N*-methyl-d-glucamine chloride (NMDG-Cl) after seal formation. All osmolalities were measured in a Wescor Vapro model 5520 osmometer (Wescor Inc., USA) calibrated by the manufacturer’s Optimol standard solutions.

### SDS-PAGE and Western blotting

Protein was isolated from cells 36 h after transfection with siRNA targeting ANO1, in a lysis buffer containing (in mM): 10 Tris–HCl, 10 EDTA, 1 % sodium dodecyl sulfate (SDS), 0.5 % sodium deoxycholate, and 1 % complete protease inhibitor mixture (Roche, Denmark), pH 7.4, and was separated by 7 % SDS-PAGE. Protein was subsequently transferred to nitrocellulose membrane (Novex, Denmark). Membranes were immunoblotted with monoclonal antibodies against ANO1 (SP31, ab64085) (Abcam, UK) or β-actin (Sigma-Aldrich, Denmark), at 1:100 and 1:3,000 dilutions, respectively, overnight at 4 °C in blocking buffer. Alkaline phosphatase-conjugated goat anti-rabbit (ANO1) or goat anti-mouse (β-actin) secondary antibodies (both Sigma-Aldrich, Denmark) were used at a 1:500 dilution in blocking buffer for 1 h at room temperature. Membranes were washed extensively in Tris-buffered saline with Tween**®** 20 (TBST) and developed using BCIP/NBT solution (Kirkegaard and Perry Laboratories, USA).

### Cell proliferation and apoptosis assay

To assess cell proliferation and apoptosis, cells were plated in duplicates on a 96-well plate and incubated in 100 μl of the cell line appropriate media described above. Cells were transfected with siRNA soon after complete attachment (approx. 3 h after seeding). Cells were treated with vehicle, 20 μM gemcitabine, 20 μM T16A_inh_-A01, or 10 μM free NS3728 (see below) after culture in a humidified 37 °C and 5 % CO_2_ incubator overnight. BrdU incorporation was measured using Cell Proliferation ELISA, BrdU (chemiluminescent) (Roche Diagnostics A/S, Denmark) following the manufacturer’s instructions. Caspases 3, 6, and 7 activation was evaluated using Homogeneous Caspases Assay (fluorimetric) (Roche Diagnostics A/S, Denmark).

### Scratch wound healing assay

Cells were seeded into Essen ImageLock 96-well plates. Cells were transfected with siRNA shortly after cells completely attached at 80 % confluency. After 24 h of incubation, confluent monolayers were scratched using the Essen 96-well WoundMaker (Essen Bioscience, USA), washed two times with the respective medium, and then incubated in medium containing 5 μM aphidicolin (Sigma-Aldrich, Denmark) and either T16A_inh_-A01, CaCC_inh_-A01, or NS3728 or the appropriate control. The wound confluence was obtained and analyzed by using the IncuCyte phase-contrast imaging and scratch wound assay system and software (Essen Bioscience, USA). Wound closure was assessed by comparing the mean relative wound density (%RWD) according to the following formula: % RWD = 100 × *w*(*t*) − *w*(0)/*c*(*t*) − *w*(*t*). Here, *w*(*t*) is the density of wounded area at time *t* and *c*(*t*) is the density of cell region at time *t*. Each experiment was conducted in triplicates.

### Measurements of the free, intracellular calcium concentration, [Ca^2+^]_i_

HPDE and BxPC-3 cells were incubated with 2 μM Fura-2 AM (Sigma-Aldrich, Denmark) for 30 min at 37 °C. Cells were subsequently washed with Krebs solution containing (in mM) 150 NaCl, 6 KCl, 1.5 CaCl_2_, 1 MgCl_2_, 10 glucose, and 10 HEPES; pH was adjusted to 7.4 using NaOH. Coverslips were mounted on a recording chamber. In the beginning of the experiment, cells were perfused with Krebs solution at 4 ml/min until baseline was stable. Thereafter, the inhibitors T16A_inh_-A01, CaCC_inh_-A01 (20 μM), or NS3728 (10 μM) were infused and cells were stimulated with general P2 receptor agonist ATP (10 μM). Changes in [Ca^2+^]_i_ were measured by a TIRF iMIC microscope (TILL Photonics, Germany). Fura-2 AM loaded cells were illuminated at 340 nm for 80 ms and 380 nm for 80 ms at 1 s intervals using a TILL Polychrome monochromator. Five hundred-nanometer emission images were captured by an image-intensifying, charge-coupled device (CCD) camera (TILL Photonics, Germany) and digitized by an image processing system (TILL Photonics, Germany). The monochromator and CCD camera were controlled by LA Live Acquisition software, which was also used for image analysis. The [Ca^2+^]_i_ was presented as the Fura-2 ratio of the fluorescence signals obtained at 340/380 nm.

### Materials and statistical analysis

2-[(5-Ethyl-1,6-dihydro-4-methyl-6-oxo-2-pyrimidinyl)thio]-N-[4-(4-methoxyphenyl)-2-thiazolyl]acetamide (T16A_inh_-A01), 6-(1,1-dimethylethyl)-2-[(2-furanylcarbonyl)amino]-4,5,6,7-tetrahydrobenzo[b]thiophene-3-carboxylic acid (CaCC_inh_-A01), and aphidicolin were obtained from Sigma-Aldrich at highest available purity. Stock solutions of 10 mM were prepared using dimethyl sulfoxide (DMSO). The VRAC and CaCC inhibitor N-[3,5-bis(trifluoromethyl)-phenyl]-N-[4-bromo-2-(1H-tetrazol-5-yl)-phenyl] urea (NS3728) was a generous gift from Palle Christophersen (NeuroSearch A/S, Denmark) (stock solution 100 mM in DMSO). Earlier reports found NS3728 to strongly bind to serum [17, 33]. We therefore used NS3728 at 50 μM for physiological assays such as proliferation, migration, and apoptosis assays, all performed in media containing serum. It is estimated that in these conditions the free inhibitor concentration amounted to 10 μM.

The results of multiple experiments are presented as means ± s.e.m., and statistical analysis was carried out by using one-way analysis of variance with Tukey’s post test or Student’s *t* test, as appropriate. *P* ≤ 0.05 was considered statistically significant.

## Results

### ANO1 is functionally overexpressed in PDAC cells and is the major mediator of CaCC current in these cells

**Fig. 1:**
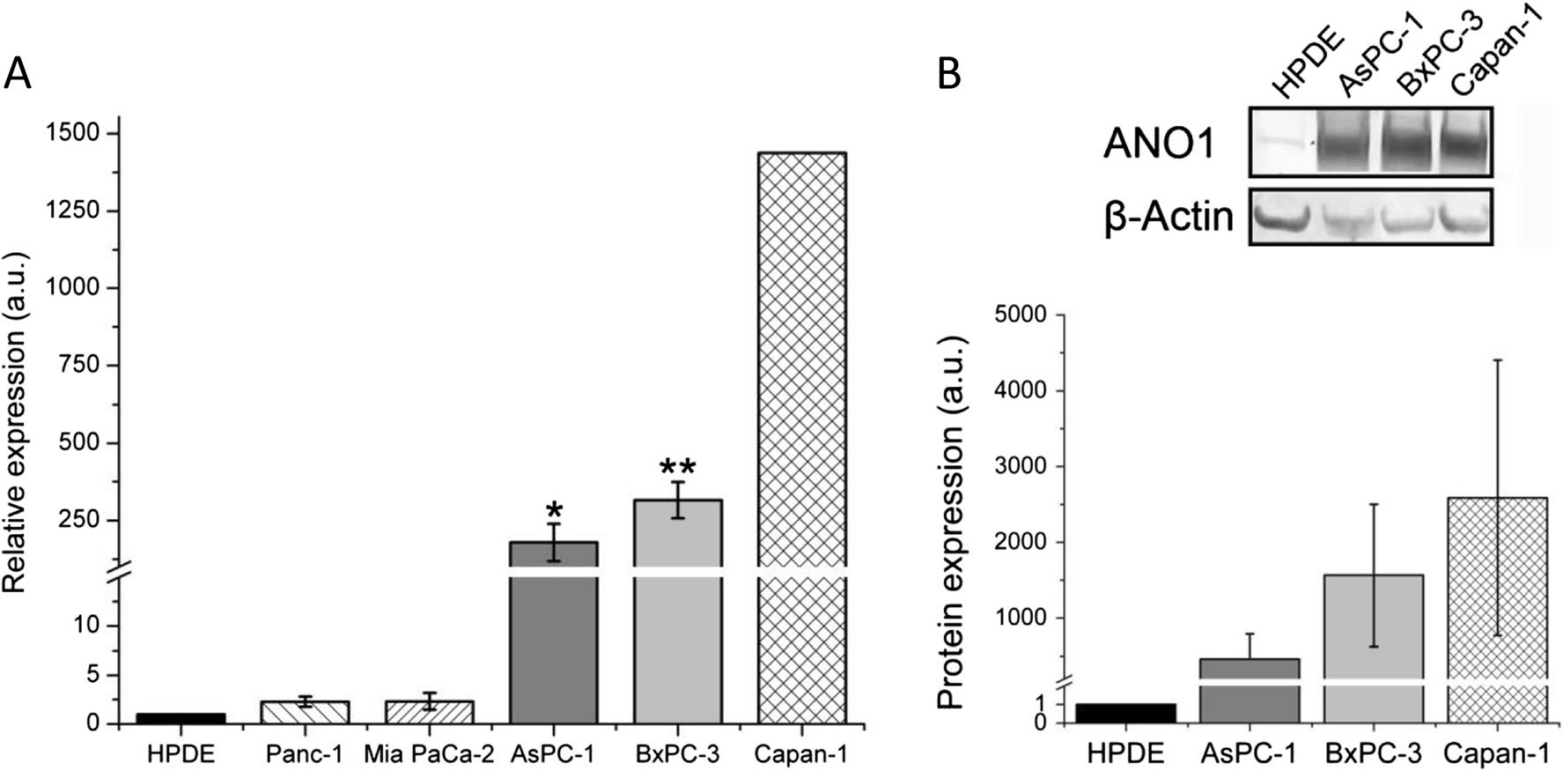
ANO1 is upregulated at the mRNA and protein levels in all PDAC cell lines tested. **a** Mean ANO1 expressions measured by RT-qPCR in five PDAC cell lines (Panc-1, Mia PaCa-2, BxPC-3, AsPC-1, and Capan-1) compared to the immortalized human pancreatic ductal cell line (HPDE). Data for Capan-1 was taken from [45] and recalculated relative to the value in the HPDE cell line. **b** Western blot analysis in three pancreatic cancer cell lines and HPDE with β-actin as a loading control. Mean ± s.e.m. of *n* = 4–5 individual experiments

To study the presence of ANO1 in PDAC cells, we evaluated the expression of ANO1 RNA by RT-qPCR in several commercially available cell lines (Mia PaCa-2, Panc-1, BxPC-3, and AsPC-1). Mia PaCa-2, Panc-1, and BxPC-3 were derived from the primary tumor of an exocrine pancreatic cancer. AsPC-1 was established from a local metastatic site in the ascites of a patient with PDAC [42]. We used a normal pancreatic ductal epithelium derived cell line (HPDE) as a control (Fig. 1). All cell lines showed an increased mRNA level, ranging from 2.3 ± 0.5- to 320 ± 60-fold over that in HPDE cells, in the Panc-1 and BxPC-3 cell lines, respectively. Our earlier study compared ANO1 mRNA levels in Capan-1 PDAC cell line to Panc-1 and CFPAC-1 [45]; using those data together with the values for HPDE and Panc-1 of the present investigation, we calculated an ∼1,450-fold upregulation of ANO1 in Capan-1 with respect to HPDE cells. Capan-1 is a liver metastasis-derived cell line [42]. We selected the aggressive pancreatic cancer cell lines AsPC-1 and BxPC-3 [6], as well as Capan-1 and HPDE cells for further experiments. To validate ANO1 expression also at the protein level in PDAC cells, we used Western blot analysis (Fig. 1b). The results showed a similar trend as observed for mRNA levels, with almost undetectable ANO1 protein expression in HPDE cells and much higher levels in the PDAC cell lines.

**Fig. 2:**
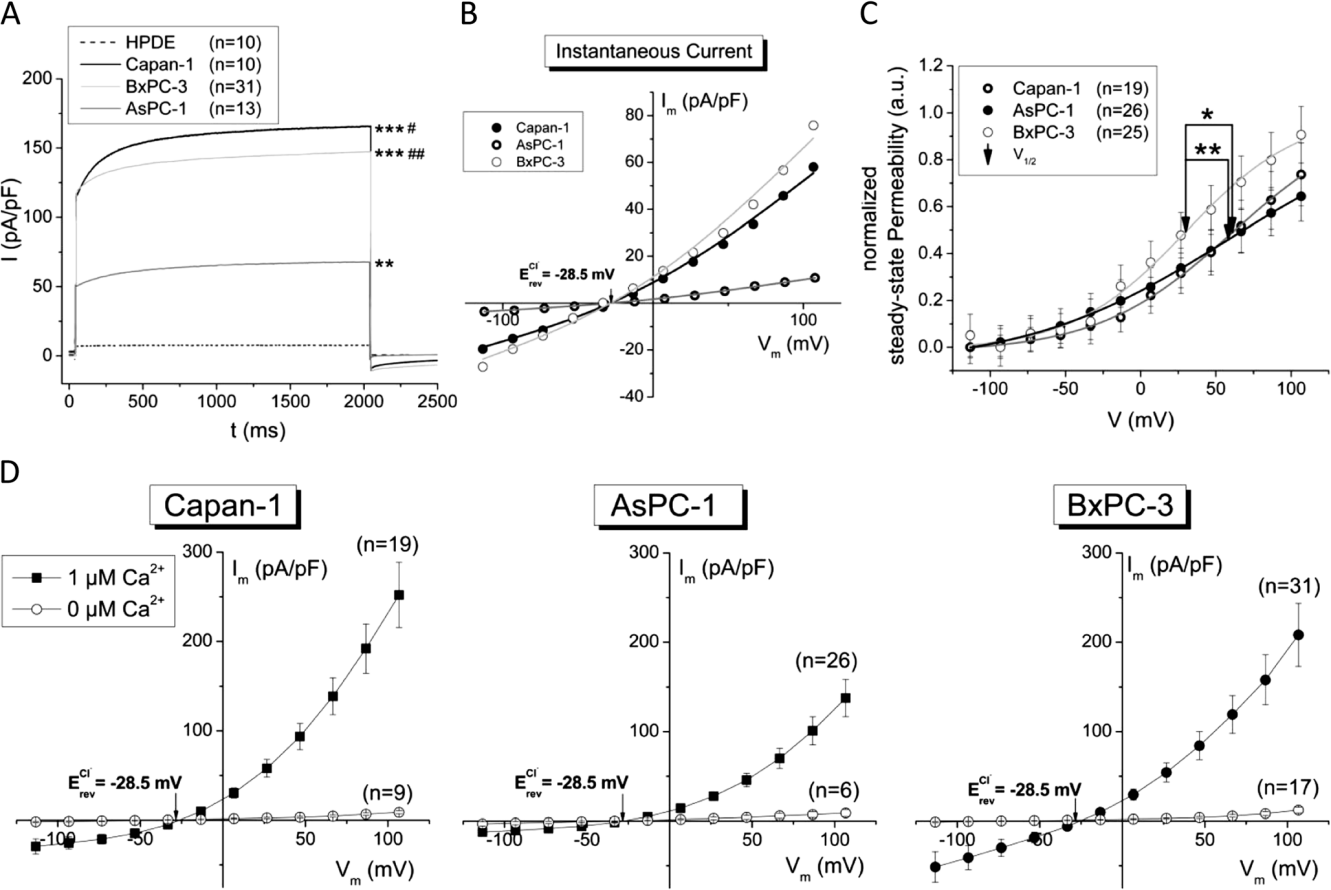
PDAC cells exhibit large Ca^2+^- and voltage-activated Cl^−^ currents that were not detected in HPDE control cells. Whole-cell patch-clamp recordings in HPDE, Capan-1, AsPC-1, and BxPC-3 cells. *V*_out_ = 0 mV and a pulse sequence from −100 to +100 mV of 20 mV increments was applied as described in “Materials and methods.” **a** Mean currents at +67 mV. Statistical analysis was made at the end of the voltage step. **b** Representative instantaneous current–voltage relationships of the different cancer cell lines fitted to the GHK current equation. **c** Normalized steady-state permeability calculated using the GHK current equation and fitted to the Boltzmann distribution. *V*_1/2_ is the voltage for half-maximal activation of the channels. **d** Steady-state *I*–*V* relationships of Capan-1, AsPC-1, and BxPC-3 cells in the presence and absence of intracellular Ca^2+^. (*n*) = number of cells measured; *asterisk*, significant when compared to HPDE (ANOVA); *number sign*, significantly different from AsPC-1 cells; *single asterisk* and *single number sign* = *p* ≤ 0.05; *double asterisk* and *double number sign* = *p* ≤ 0.01; *triple asterisk* and *triple number sign* = *p* ≤ 0.001

The functional expression of the protein in the plasma membrane was assessed using the whole-cell patch-clamp technique (Fig. 2). The time-dependent current activation was recorded as described in “Materials and methods”. Mean traces at *V*_m_ = +67 mV with 1 μM intracellular Ca^2+^ revealed a large voltage-dependent current with activation at positive membrane potentials in all cancer cell lines. This was most pronounced in Capan-1 cells and barely detectable in HPDE control cells (Fig. 2a). The instantaneous currents of the three cancer cell lines followed GHK rectification (Fig. 2b). It was thus possible to analyze steady-state permeability’s using the GHK current equation (Fig. 2c). The instantaneous current–voltage curve of a few cells reversed at far more positive potentials as the calculated equilibrium potential for Cl^−^, those cells were omitted from the calculation of permeability. Capan-1 and AsPC-1 cells showed similar activation curves with half-maximal activation of the channels (*V*_1/2_) at 61 ± 8 and 58 ± 8 mV, respectively. This is in good agreement with earlier studies in ANO1-transfected HEK293 cells [47]. BxPC-3 cells exhibited a significantly shifted permeability—voltage curve with *V*_1/2_ = 30 ± 8 mV. This altered gating in BxPC-3 cells may be due to differential expression of other possible modulators (e.g., calmodulin) or mutations in the voltage- or Ca^2+^-sensing first intracellular loop [47]. Further studies are needed to investigate this phenomenon. Steady-state current–voltage curves showed an outward-rectifying current with a reversal potential (*V*_Rev_) close to the Cl^−^ equilibrium potential (E_Cl_, Fig. 2d). Removal of Ca^2+^ from the pipette solution almost completely eliminated this current in the cancer cell lines (Fig. 2d). These biophysical properties match the characteristics described for ANO1 [48].

**Fig. 3:**
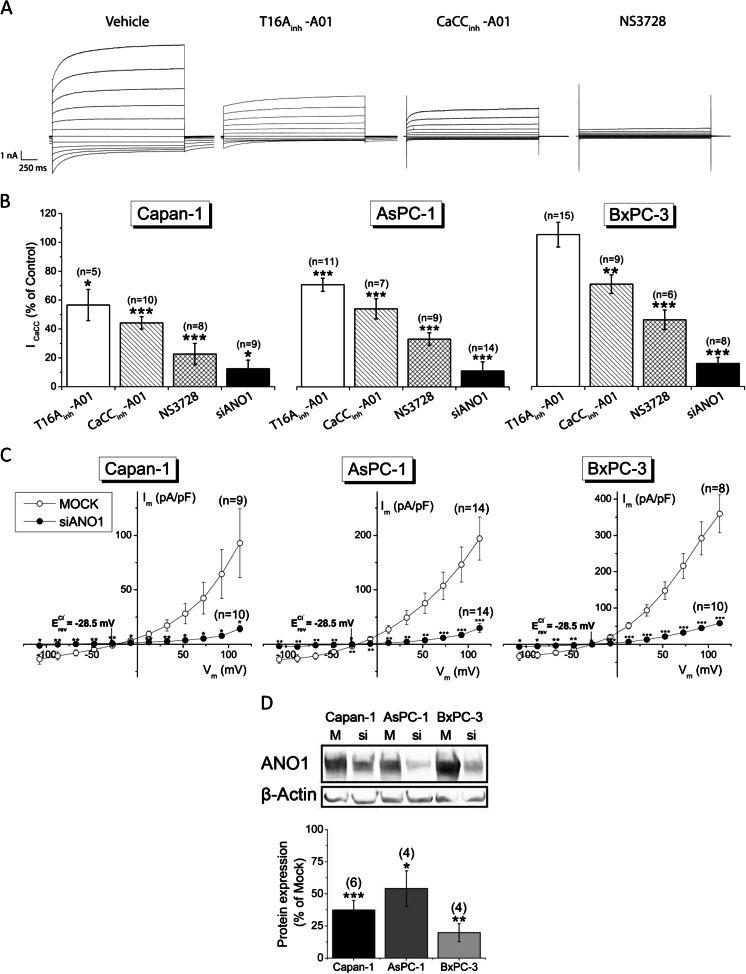
ANO1 is the major contributor of CaCC currents in PDAC cells and is sensitive to Cl^−^ channel inhibitors and siRNA. Whole-cell patch-clamp recordings with 1 μM Ca^2+^ in pipette solution. **a** Representative current traces measured in Capan-1 cells upon exposure to different inhibitors. **b** Quantification of CaCC currents at +67 mV recorded with inhibitors compared to currents in presence of DMSO; currents of siANO1 cells were compared to those of MOCK transfected cells. **c** Steady-state *I*–*V* relationships of cells transfected with either scrambled siRNA (MOCK) or siRNA targeting ANO1. **d** Densitometric quantification of Western blot analysis of siRNA knockdown. β-Actin was used as a loading control. (*n*) = number of individual experiments

To quantify the ANO1-mediated part of the total CaCC current, we used siRNA knockdown, as well as the three Cl^−^ channel inhibitors NS3728 (10 μM), CaCC_inh_-A01 (20 μM), and T16A_inh_-A01 (20 μM). NS3728 is an inhibitor of VRAC but was also found to inhibit CaCC [17]. CaCC_inh_-A01 is known to potently inhibit CaCC current but was further shown to alter [Ca^2+^]_i_ signals [18]. T16A_inh_-A01 is the latest developed inhibitor of ANO1; no unspecific effects have been identified so far. Results are shown in Fig. 3. Application of T16A_inh_-A01 attenuated the CaCC current significantly in both Capan-1 (43 ± 8 % inhibition at +67 mV) and AsPC-1 (29 ± 2 %) cells, but was ineffective on BxPC-3 cells (Fig. 3b). CaCC_inh_-A01 inhibited the current more effectively in all tested cell lines with 56 ± 6 % inhibition in Capan-1, 46 ± 6 % in AsPC-1, and 29 ± 3 % in BxPC-3 cells, respectively (all calculated at +67 mV). Application of NS3728 resulted in the most pronounced inhibition in all cell lines with 77 ± 26 % in Capan-1, 67 ± 9 % in AsPC-1, and 54 ± 8 % in BxPC-3 cells at +67 mV. Using siRNA targeting ANO1, the CaCC currents decreased by 88 ± 42 % in Capan-1, 89 ± 51 % in AsPC-1, and 84 ± 23 % in BxPC-3 cells when compared to currents measured in cells transfected with scrambled siRNA (MOCK) (Fig. 3c). Successful knockdown of ANO1 protein expression was confirmed using Western blot (Fig. 3d). Protein expression was decreased to 37 ± 8 % in Capan-1, 54 ± 14 % in AsPC-1, and 20 ± 7 % in BxPC-3 cells.

### All tested cells exhibit a large volume-regulated anion current

**Fig. 4:**
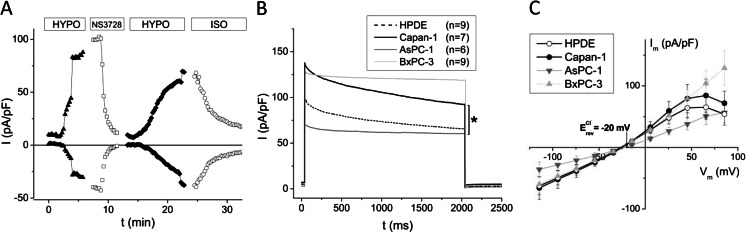
All tested cell lines exhibit large swelling-activated Cl^−^ currents. Response of whole-cell currents to hypotonic bath solution; pipette solution was 0 μM Ca^2+^ with 10 mM EGTA. **a** Time course of currents in Capan-1 cells as described in “Materials and methods.” Currents at −47 and +93 mV were corrected for liquid junction potential, *n* = 24 cells. Rate of activation varied among cells. **b** Currents at +65 mV, measured using the voltage protocol described in legend of Fig. 2. **c** Steady-state *I*–*V* relationships given as mean ± s.e.m. (*n*) = number of cells

The above results provide compelling evidence that ANO1 carries the major fraction of CaCC in PDAC cells. In order to further characterize the role of Cl^−^ channels in PDAC cells, we next evaluated the role of the volume-regulated anion channel (VRAC or LRRC8), the genetic identity of which was recently identified [34, 44]. We studied VRAC phenotype by whole-cell voltage-clamp recordings of cells exposed to a hypotonic bath solution and results are shown in Fig. 4. To rule out contributions from CaCC, Ca^2+^ was omitted from the pipette solution and 10 mM EGTA was included. A bath solution change from 315 to 210 mosmol/kg (removal of d-mannitol) caused the cells to swell and evoked a large current that was completely inhibited upon application of 10 μM of NS3728 (Fig. 4a). Both the activation by volume expansion and the NS3728-mediated inhibition were reversible. The time course of swelling activation varied significantly among individual cells. However, large VRAC currents were detected in all tested cell lines with most prominent currents seen in Capan-1 and BxPC-3 cells (Fig. 4b). We also observed VRAC current inactivation at *V*_m_ > 0 mV with flat tail currents at depolarized membrane potentials, as also reported previously [30]. This voltage dependency of *I*_VRAC_ was pronounced in HPDE and Capan-1 cells; BxPC-3 and AsPC-1 cells showed weaker inactivation at positive voltages (Fig. 4b, c). It was recently suggested that VRAC is a heteromer formed by different subtypes of the LRRC8 protein family, which exhibit different gating at positive potential [44]. The apparent difference in gating between the cell lines might be due to differential expression of the different LRRC8 subtypes. However, further studies are required to confirm this. The *V*_Rev_ of the activated current was −25.7 ± 0.7 mV (HPDE), −24 ± 2 mV (Capan-1), −24 ± 2 mV (AsPC-1), and 28 ± 3 mV (BxPC-3), as compared to the calculated Cl^−^ equilibrium potential of −20 mV (Fig. 4c).

### T16A_inh_-A01 and CaCC_inh_-A01 do not inhibit VRAC; NS3728 is a potent inhibitor of both VRAC and ANO1

**Fig. 5:**
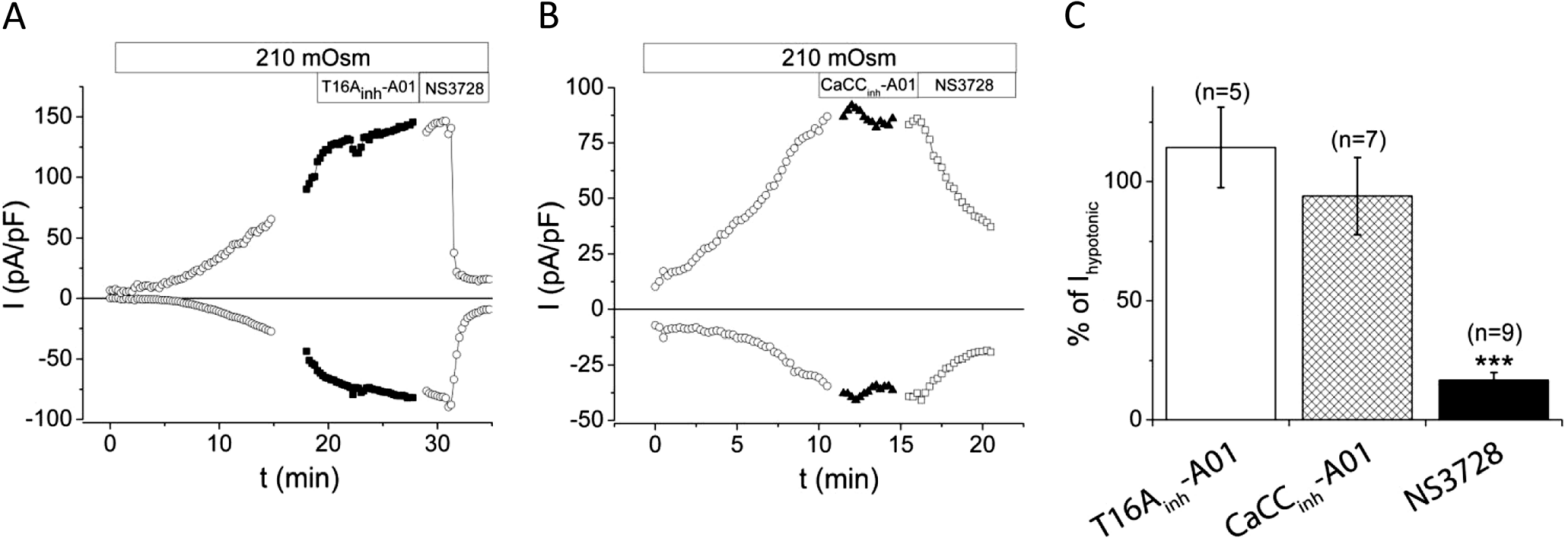
T16A_inh_-A01 and CaCC_inh_-A01 do not inhibit VRAC. **a**, **b** Representative current densities over time in HPDE and BxPC-3 cells, respectively, exposed to hypotonic bath solution and pipette solution containing 0 μM Ca^2+^. Inhibitors were applied for 3–10 min when the maximum volume-activated current was obtained. **c** Quantification of the effect shown in percent of maximum VRAC current at +65 mV. Data shown are mean ± s.e.m.

**Fig. 6:**
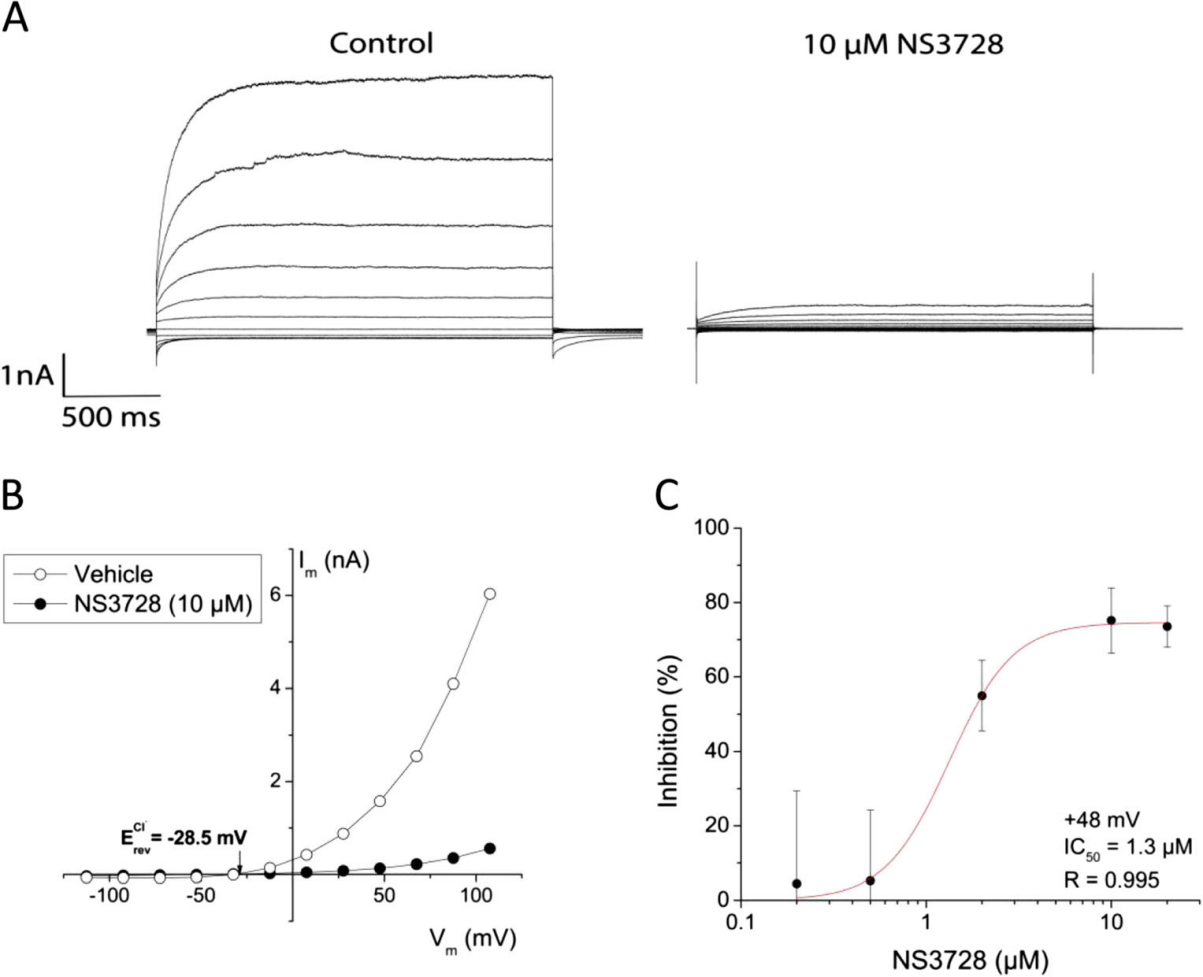
NS3728 inhibits ANO1. Whole-cell currents of HEK293 cells transiently transfected with mANO1-GFP with 1 μM free Ca^2+^ in pipette solution. **a** Currents recorded using the step protocol with and without NS3728 (10 μM) **b** *I*–*V* relationships. Cl^−^ equilibrium potential, *E*_Cl_ = −28.5 mV. **c** Dose–response curve of NS3728 at *V*_m_ = +48 mV. The *solid line* is the fit by the Hill equation (*n* = 3–8 cells, depending on concentration)

In order to evaluate the inhibitors of the two Cl^−^ channels, we studied effects of the ANO1 inhibitors, T16A_inh_-A01 and CaCC_inh_-A01, and of the VRAC inhibitor, NS3728, on volume-induced currents. Cells were exposed to hypotonic solution in the absence of intracellular Ca^2+^, and after full activation of the membrane current, the HPDE and BxPC-3 cells were exposed to 20 μM T16A_inh_-A01 or CaCC_inh_-A01 followed by NS3728 (Fig. 5a, b). Statistical analyses of currents were performed at *V*_m_ = +65 mV before and after application of the respective inhibitor (Fig. 5c). Neither T16A_inh_-A01 nor CaCC_inh_-A01 inhibited the VRAC currents significantly. In contrast, NS3728 decreased the fully activated VRAC current to 17 ± 4 % of that in the absence of the inhibitor. NS3728 was originally found to inhibit VRAC [12], but it also inhibited CaCC (Fig. 3) as also shown in another study [17]. To elucidate a possible effect of NS3728 on ANO1, we used HEK293 cells transiently transfected with mANO1-GFP as described recently [10]. Transfection with ANO1-GFP resulted in induction of a Ca^2+^- and voltage-dependent, outwardly rectifying current (Fig. 6a) with a *V*_rev_ near *E*_Cl_ (Fig. 6b). These currents were almost completely inhibited when treated with NS3728 (10 μM free concentration). The associated IC_50_ calculated at *V*_m_ = +48 mV was estimated to be 1.3 μM (Fig. 6c).

### ANO1 plays no role in PDAC cell proliferation; VRAC does

**Fig. 7:**
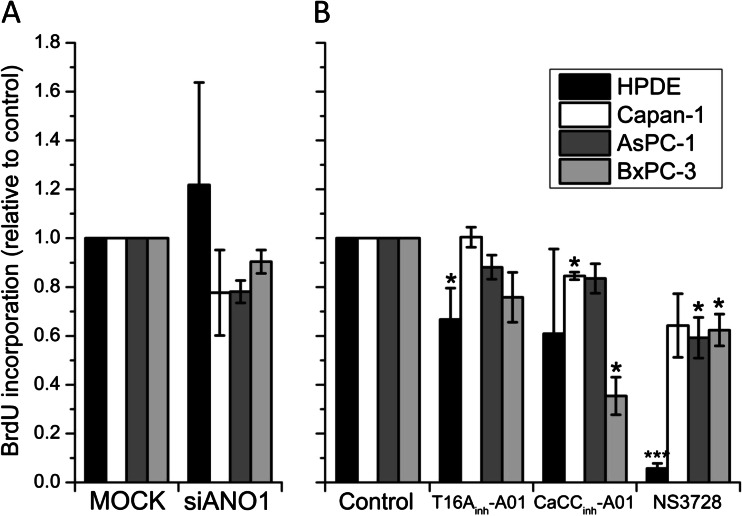
VRAC, but not ANO1, regulates cellular proliferation. **a** Summary of BrdU incorporation in HPDE, Capan-1, AsPC-1, and BxPC-3 cells after various treatments as indicated. Cells were transfected shortly after complete attachment (approx. 3 h) and BrdU incorporation was assessed 36 h after transfection. To investigate effect of inhibitors, cells were incubated for 24 h with respective inhibitor. Values shown are relative to appropriate control. Data shown represents mean ± s.e.m. of *n* = 4–6 individual experiments. **p ≤ 0.01 and ***p ≤ 0.001 when compared with control

To assess the roles of ANO1 and VRAC in cell proliferation, we measured BrdU incorporation in mock (5 nM) and ANO1 siRNA (50 nM) transfected cells, as well as in the presence or absence of T16A_inh_-A01 (20 μM), CaCC_inh_-A01 (20 μM), NS3728 (10 μM free concentration), and the standard chemotherapeutical drug gemcitabine (20 μM, not shown) (Fig. 7). siRNA transfection targeting ANO1 did not cause any significant change in proliferation in any of the tested cell lines (Fig. 7a). This result indicates, quite unexpectedly, that ANO1 plays no major role in cell cycle progression. In agreement with this, none of the ANO1-overexpressing cancer cells showed any significant decrease in proliferation in response to T16A_inh_-A01 (Fig. 7b). A slight, but non-significant decrease of proliferation was seen in BxPC-3 cells after T16A_inh_-A01 treatment, although T16A_inh_-A01 did not affect CaCC currents in these cells in patch-clamp recordings. Also HPDE cells, which do not exhibit great CaCC (Fig. 2a), showed inhibited proliferation with T16A_inh_-A01. The more potent inhibitor CaCC_inh_-A01 decreased proliferation in Capan-1 slightly and had larger effect on BxPC-3 cells. Capan-1 cells that reacted most prominently to CaCC_inh_-A01 (56 ± 6 % inhibition) in patch-clamp measurements showed only a slight decrease in proliferation after addition of the inhibitor.

**Fig. 8:**
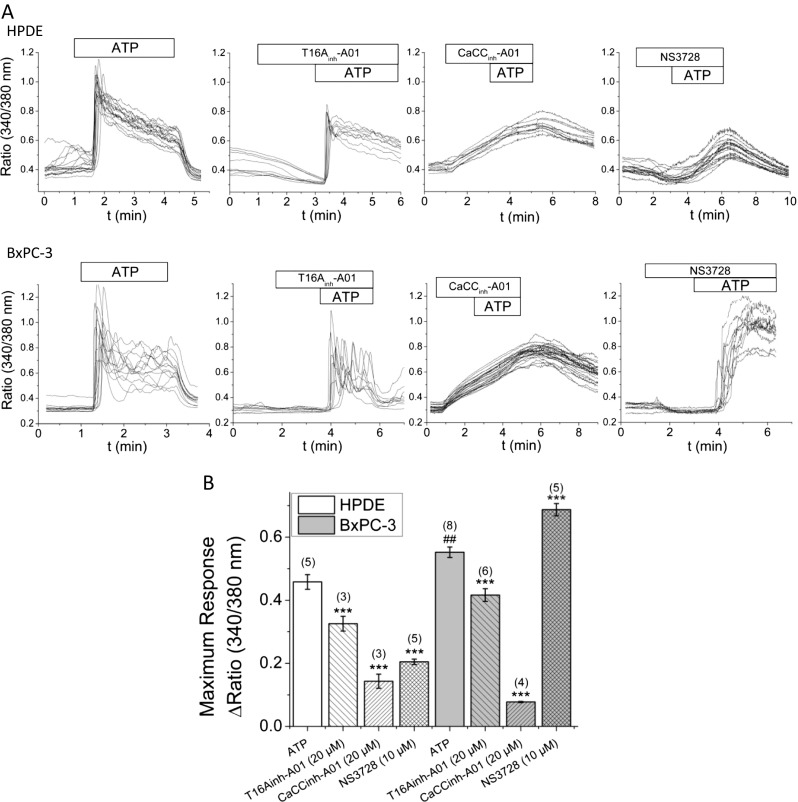
Cl^−^ channel inhibitors alter ATP-induced Ca^2+^ signals. Intracellular Ca^2+^ measurements in FURA-2 loaded HPDE and BxPC-3 cells. **a** Representative single-cell traces of individual experiments. Different substances were applied as indicated. ATP was applied at 10 μM, T16A_inh_-A01 and CaCC_inh_-A01 at 20 μM, and NS3728 at 10 μM. **b** Summary of maximum ATP-induced changes in [Ca^2+^]_i_; for CaCC_inh_-A01, these values may be overestimates as the peak response was not present. (*n*) = number of individual experiments; each containing between 5–45 single cells.***p ≤ 0.001 when compared to no inhibitor condition; ^##^ *p* ≤ 0.01 when compared with HPDE no inhibitor

Most importantly, Fig. 7b also shows that application of NS3728 resulted in a pronounced decrease in cellular proliferation in all cells. The most drastic effect was observed in HPDE control cells. The very low expression level of ANO1 in this cell line suggests that the inhibition of proliferation by NS3728 is not ANO1-mediated, but rather reflects inhibition of VRAC or altered [Ca^2+^]_i_ signaling (Fig. 8). In agreement with this, it is long known that VRAC is involved in progression throughout the cell cycle of different cancer types [17, 40]. As expected, gemcitabine treatment caused an almost complete inhibition of proliferation in all tested cells (data not shown).

### All Cl^−^ channel inhibitors alter intracellular Ca^2+^ signals

Activation of CaCC requires an increase in [Ca^2+^]_i_, which can be achieved by stimulation of receptors such as purinergic receptors. Therefore, we studied the effect of Cl^−^ channel inhibitors on changes in [Ca^2+^]_i_ induced by purinergic signaling. We chose HPDE cells with very low expression levels of ANO1 and the highly ANO1 overexpressing cell line BxPC-3 for investigation. Fura-2 loaded HPDE and BxPC-3 cells were stimulated with ATP (10 μM) (Fig. 8) or exposed to the inhibitor prior to stimulation. ATP stimulation caused a 0.46 ± 0.03 peak increase in the Fura-2 ratio in HPDE and 0.55 ± 0.02 in BxPC-3 cells. Cells first exposed to T16A_inh_-A01 showed a significantly attenuated peak response to ATP resulting in a 0.32 ± 0.03 (HPDE) and 0.42 ± 0.03 (BxPC-3) change in Fura-2 ratio (Fig. 8). However, no effect of the inhibitor on basal Ca^2+^ level was observed. Exposure to CaCC_inh_-A01 (20 μM) caused a reversible increase in [Ca^2+^]_i_ in both cell lines tested. Subsequent application of ATP elicited no additional effect on top of inhibitor-induced [Ca^2+^]_i_ increase. No significant change in basal [Ca^2+^]_i_ was observed in HPDE and BxPC-3 cells when exposed to NS3728 (10 μM). Pretreatment with NS3728 caused a significantly attenuated response in HPDE cells (0.21 ± 0.01 Δratio 340/380 nm). In contrast, in BxPC-3 cells, NS3728 pretreatment elicited a prolonged increase in [Ca^2+^]_i_ upon ATP stimulation. The responses are summarized in Fig. 8b.

### ANO1 is crucial for cell migration

**Fig. 9:**
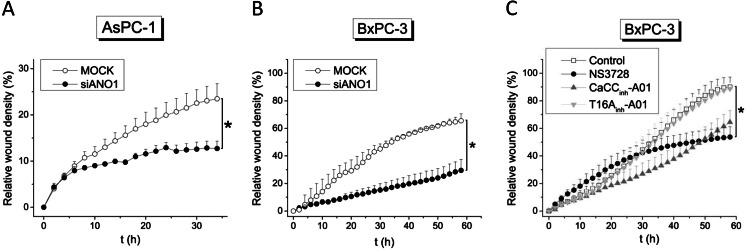
ANO1 supports migration of PDAC cells. Wound healing assay is shown as relative wound density (density of wounded area relative to confluency of cell region) over time. Aphidicolin (5 μM) was added to stop proliferation. **a** AsPC-1 cells and **b** BxPC-3 cells after transfection with either scrambled siRNA (5 nM) or siRNA targeting ANO1 (50 nM). **c** BxPC-3 cells in presence of different inhibitors. Data shown represents mean ± s.e.m. of *n* = 4 experiments; each run was carried out in triplicates

Earlier studies already revealed ANO1 as a crucial player in cell migration [15, 35]. We therefore assessed a possible contribution of ANO1 in PDAC cell migration using wound healing assay. Capan-1 cells did not show any wound closure in this time frame and were therefore not investigated. The experiments were conducted in the presence of 5 μM aphidicolin to rule out a possible effect on cell proliferation. Figure 9a shows relative wound density (see Materials and Methods) over time, monitored in AsPC-1 cells transfected with either scrambled siRNA (5 nM)(MOCK) or siRNA targeting ANO1 (50 nM). ANO1 knockdown cells showed a significantly decreased migration (Fig. 9a). However, the relative wound density curve over time saturated after 36 h and only partial wound closure (24 ± 4 %) was reached in the control situation. AsPC-1 cells were therefore not well suited for studies of lateral motility. BxPC-3 cells showed a much more rapid wound closure with complete closure after 60 h in most of the control experiments. Migration was significantly decreased to 30 ± 8 % after 60 h after transfection with siANO1 (Fig. 9b). In another series of experiments, we exposed the cells to T16A_inh_-A01 (20 μM), CaCC_inh_-A01 (20 μM), and NS3728 (10 μM free concentration) (Fig. 9c). Application of T16A_inh_-A01 had no detectable effect on migratory behavior. CaCC_inh_-A01 seemed to slow down wound closure; however, this did not reach significance. When exposed to NS3728, an initial acceleration was observed that began to saturate after 30 h and resulted in 54 ± 8 % relative wound density after 60 h. All inhibitors were applied at several orders of magnitude above the IC_50_ value for ANO1, yet none caused effects on wound closure comparable to those observed in the knockdown cells. These results are in good agreement with whole-cell patch-clamp recordings of CaCC currents, in which ANO1 knockdown also showed the strongest inhibitory effect (Fig. 3).

## Discussion

In the present study, we provided compelling evidence that ANO1 is functionally overexpressed in human pancreatic cancer cell lines and that it carries the major component of CaCC currents in these cells. Below, we discuss the possible roles of ANO1 in PDAC cell behavior, as well as some unexpected effects of putative ANO1 inhibitors.

### All Cl^−^ channel inhibitors inhibit CaCC but also have other side effects

siRNA knockdown of ANO1 almost completely abolished the CaCC-mediated current in all cells tested. Surprisingly, to date, the most specific inhibitor of ANO1, T16A_inh_-A01 [24], did not inhibit the CaCC current in BxPC-3 cells, even when applied several orders of magnitude above the IC_50_ value for ANO1 (20 μM) [24]. Inhibition was observed in other PDAC cells, but efficacy was still only moderate (Capan-1 and AsPC-1 cells) (Fig. 3). A recent study by Scudieri et al. of ANO1 in bronchial epithelial cells reports similar observations [39]. They found that siRNA targeting ANO1 diminished Ca^2+^-activated Cl^−^ secretion almost entirely, yet T16A_inh_-A01 (10 μM) showed relatively weak inhibition. Moreover, these researchers found that cells prior exposed to T16A_inh_-A01 (10 μM) had significantly attenuated UTP-induced Cl^−^ secretion in short-circuit currents recordings. This is in line with our measurements of attenuated ATP-induced increase in [Ca^2+^]_i_ in the presence of this inhibitor (Fig. 8). We also observed off-target effects of T16A_inh_-A01 when applied in proliferation experiments. The inhibitor resulted in a significant inhibition of proliferation in HPDE cells, which almost completely lack ANO1 (Figs. 1, 2, and 7) and yet have attenuated response to ATP-induced [Ca^2+^]_i_ signals. Taken together, these results suggest that T16A_inh_-A01 is not suited as a specific inhibitor of ANO1 and that its mechanism of action might either be more indirect or may depend on the type of cell, ANO1 isoforms and/or experimental conditions.

CaCC_inh_-A01 inhibited CaCC current stronger in all cell lines tested (Fig. 3). No effect of CaCC_inh_-A01 on VRAC was observed (Fig. 5b). However, we revealed large unspecific effects of the inhibitor on [Ca^2+^]_i_ which slowly increased with the inhibitor, ATP stimulation had no further effects, and the effects were fully reversible (Fig. 8). Similar unspecific effects of the inhibitor were also observed by others [18]. CaCC_inh_-A01 was further reported to inhibit other CaCCs [18].

The third inhibitor used in this investigation, NS3728, also has limited specificity. It is an inhibitor of VRAC and ANO1 (Figs. 4 and 6), but may also inhibit other molecular entities involved in CaCC [17]. We further found that NS3728 altered ATP-induced [Ca^2+^]_i_ signals in a cell line-dependent fashion (Fig. 8), such that PDAC cells were even more responsive to ATP. Our study thus illustrates that presently available inhibitors are problematic and more selective inhibitors are still required.

### ANO1 has no role in cell proliferation but supports migration

The physiological role of CaCC (e.g., ANO1) in pancreatic ducts is in mediating secretion [25, 45]. One of the interesting questions is how overexpression of this channel could regulate other cellular functions in PDAC. We found no correlation between ANO1 protein level and cell proliferation (Figs. 1 and 7) or effector caspase activation after gemcitabine treatment (online resources Fig. S1) in any of the tested cells. Our findings are in agreement with recent reports on head and neck squamous cell carcinoma cells, where no correlation between ANO1 expression and channels function (siRNA knockdown or CaCC_inh_-A01) and cell proliferation was found [1, 35]. However, these results stand in contrast to reports in which ANO1 was shown to play a pro-proliferative role in cancer cells. These studies use different approaches covering xenograft models [7], RNAi knockdown [22], as well as small-molecule inhibitors [23]. The latter study was entirely based on T16A_inh_-A01 and uses the pancreatic cancer cell line CFPAC-1, which has also a defect in another Cl^−^ channels (i.e., CFTR). Unspecific effects of the inhibitor as shown in the present investigation may explain these results. However, the function of ANO1 in cell proliferation may depend on cell environment, cell type, or other factors. The most significant decrease in proliferation of PDAC cell lines was observed with NS3728 exposure, an effect which can probably be assigned to inhibition of VRAC and cell volume regulation. In addition, altered [Ca^2+^]_i_ signaling, as shown in Fig. 8, may also contribute to NS3728-mediated inhibition of cell proliferation. Taken together, no correlation between ANO1 expression and PDAC cell proliferation was observed in the present study. However, our results once again highlight the importance of VRAC in proliferation previously shown in several cell types [13].

Most importantly, we found that siRNA knockdown of ANO1 resulted in a significantly decreased migratory rate in both AsPC-1 and BxPC-3 cells (Fig. 9). This pro-migratory role of ANO1 was further confirmed by pharmacological inhibition of the channel. Thus, CaCC_inh_-A01 and NS3728 caused a decrease in migration in BxPC-3 cells, whereas T16A_inh_-A01 was found to be ineffective. These results correlate directly with the inhibitory potential of these inhibitors on CaCC current (Fig. 3b), suggesting that ANO1 functions as an ion channel which has a crucial role in supporting migration. These results fit with the commonly accepted model for cell migration, where cell shape changes caused by local volume changes are driven by fluxes of K^+^ and Cl^−^ ions and accompanied by H_2_O [38]. We and others provided compelling evidence that ANO1 is involved in the migratory machinery in other cell types [15, 35]. On the other hand, Capan-1 cells, which showed the highest functional ANO1 expression in the present study, migrate very slowly, whereas HPDE cells which are almost devoid of ANO1 showed fast wound closure in a scratch assay (data not shown). This indicates that whereas Cl^−^ currents are likely a prerequisite to cell migration, the current need not be carried by ANO1. In HPDE cells, VRAC may be a possible candidate.

Our study points out that to further target the functions of ANO1 and VRAC in cancers overexpressing these currents, development of more selective small-molecule inhibitors would be useful. In conclusion, our study shows that ANO1 is functionally overexpressed in PDAC cells and its role is related to cell migration but not to proliferation. ANO1 may therefore pose a good target for control of metastatic potential of PDAC.

## Electronic supplementary material

Below is the link to the electronic supplementary material.

ESM 1 (PDF 5173 kb)
