# Supplementary material for: ANO1 (TMEM16A) in pancreatic ductal adenocarcinoma (PDAC)
Source: Pflugers Arch. 2014 Aug 28;467(7):1495–508. doi: 10.1007/s00424-014-1598-8 (PMC4464647; doi:10.1007/s00424-014-1598-8)
Supplement: Supplementary file 1 — (PDF 5173 kb) [file 424_2014_1598_MOESM1_ESM.pdf]

Online rescouces

ANO1 (TMEM16A) in Pancreatic ductal adenocarcinoma (PDAC)
Pflügers Archiv - European Journal of Physiology

Sauter DRP[1], Novak I[2], Pedersen SF[1], Larsen EH[2], Hoffmann EK[1]
Correspondence: Daniel R.P. Sauter; Department of Biology; August Krogh Building; Universitetsparken 13; 2100 Copenhagen Ø; Denmark; E-mail: daniel.sauter@bio.ku.dk

S1

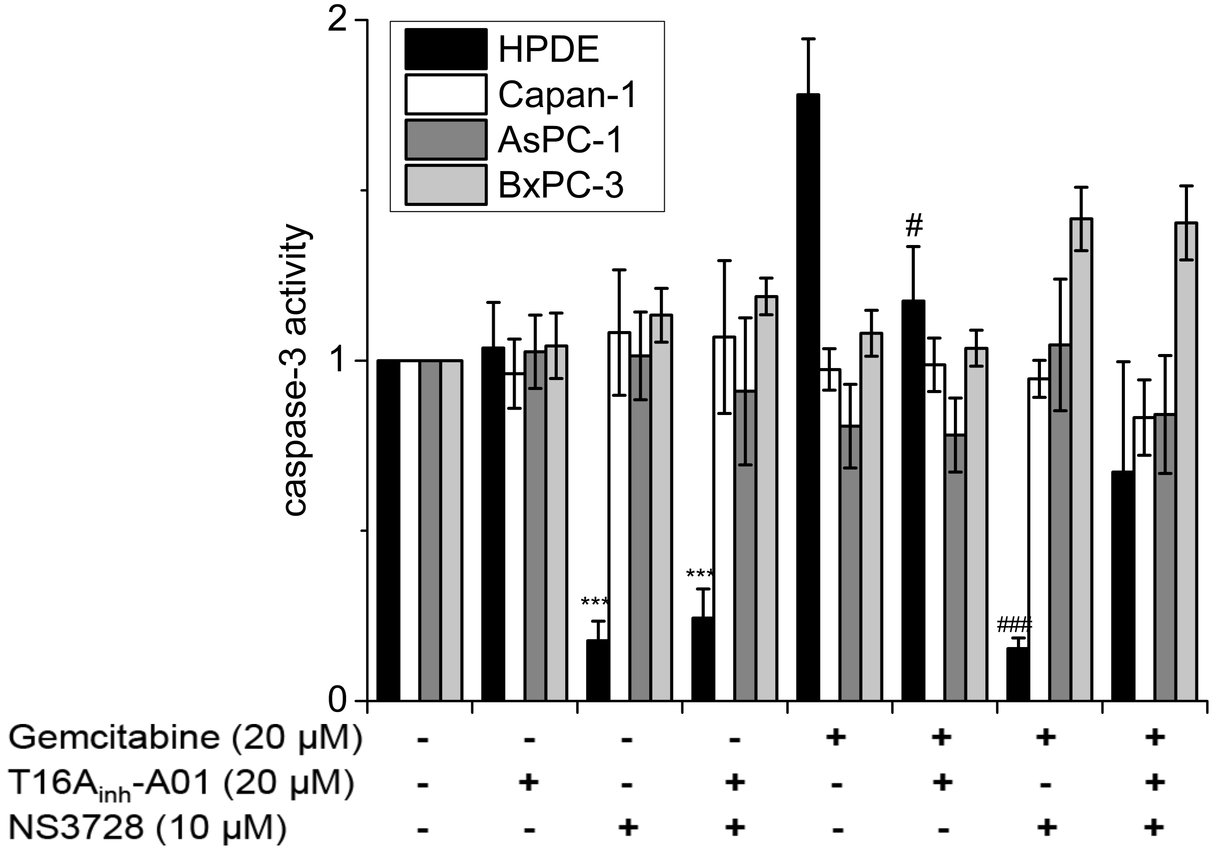

Fig. S1: NS3728 protects from activated caspases after Gemcitabine treatment. Mean Caspase-3 activity in HPDE, Capan-1, AsPC-1 and BxPC-3 cells after various treatments as indicated relative to the vehicle treated cells (n=4-5). Cells were exposed to respective inhibitor for 24 h before Caspase-3 activity was measured. NS3728 significantly protected HPDE control cells from undergoing apoptosis and was further able to protect from Gemcitabine induced apoptosis. This inhibition was observed less pronounced after application of $T16A_{inh}$-A01 but did not reach significance. * indicated significant response when compared to control; #, significantly different from Gemcitabine treated cells; * and # = p ≤0.05; *** and ### = p ≤0.001.
